# Differential Canalograms Detect Outflow Changes from Trabecular Micro-Bypass Stents and Ab Interno Trabeculectomy

**DOI:** 10.1038/srep34705

**Published:** 2016-11-04

**Authors:** Hardik A. Parikh, Ralitsa T. Loewen, Pritha Roy, Joel S. Schuman, Kira L. Lathrop, Nils A. Loewen

**Affiliations:** 1 Department of Ophthalmology, University of Pittsburgh School of Medicine, Pittsburgh, PA 15213, United States; 2 New Jersey Medical School, Rutgers State University of New Jersey, Newark, NJ 07103, United States; 3 Department of Ophthalmology, New York University School of Medicine, NY 10016, United States; 4 Department of Bioengineering, University of Pittsburgh Swanson School of Engineering, Pittsburgh, PA 15261, United States

## Abstract

Recently introduced microincisional glaucoma surgeries that enhance conventional outflow offer a favorable risk profile over traditional surgeries, but can be unpredictable. Two paramount challenges are the lack of an adequate training model for angle surgeries and the absence of an intraoperative quantification of surgical success. To address both, we developed an *ex vivo* training system and a differential, quantitative canalography method that uses slope-adjusted fluorescence intensities of two different chromophores to avoid quenching. We assessed outflow enhancement by trabecular micro-bypass (TMB) implantation or by ab interno trabeculectomy (AIT). In this porcine model, TMB resulted in an insignificant (p > 0.05) outflow increase of 13 ± 5%, 14 ± 8%, 9 ± 3%, and 24 ± 9% in the inferonasal, superonasal, superotemporal, and inferotemporal quadrant, respectively. AIT caused a 100 ± 50% (p = 0.002), 75 ± 28% (p = 0.002), 19 ± 8%, and 40 ± 21% increase in those quadrants. The direct gonioscopy and tactile feedback provided a surgical experience that was very similar to that in human patients. Despite the more narrow and discontinuous circumferential drainage elements in the pig with potential for underperformance or partial stent obstruction, unequivocal patterns of focal outflow enhancement by TMB were seen in this training model. AIT achieved extensive access to outflow pathways beyond the surgical site itself.

Advances in glaucoma surgery device engineering in the sub-millimeter range and improved surgical techniques have caused a rapid evolution of microincisional glaucoma surgeries (MIGS)^1^. Compared to traditional trabeculectomies and tube shunts^2^, these procedures are significantly faster and have a favorable risk profile^1^,^3^. This also allows combining procedures^3^ even in complex scenarios^4^,^5^. The procedures described here, a trabecular meshwork micro-bypass stent (TMB, iStent^®^ G1, Glaukos Corporation, Laguna Hills, CA, USA) and trabectome-mediated ab interno trabeculectomy (AIT, trabectome, Neomedix, Tustin, California, USA) are the only MIGS approved by the Food and Drug Administration of the United States (USFDA). The TMB is a heparin-coated titanium stent measuring 1 × 0.3 mm that is inserted through the trabecular meshwork (TM) into Schlemm’s canal^6^. AIT is a plasma surgery ablation technique that uses a 550 kHz bipolar electrode tip to remove the TM^1^. Ablation is performed over 90 to 180 degrees, allowing to tap into several drainage segments in comparison to single-access MIGS procedures^7^.

The main shortcoming of both procedures is that they are relatively difficult to master and that surgical success of bypassing or removing the TM depends on a functioning downstream collector channel system. There is currently no method to choose the surgical site based on where reduced flow areas are. Similarly, there is no way to precisely locate where the remaining outflow resistance resides in patients where outflow enhancement fails^3^ despite an otherwise correct surgical technique that should have eliminated the primary resistance of the TM^8^,^9^. Fellman *et al*. described that upon careful observation a successful TM ablation correlates with change of the episcleral fluid wave and outcomes of AIT^10^.

Past studies have quantified the bulk outflow of aqueous humor^11^ or modeled focal outflow mathematically^12^. Tracers, such as cationized ferritin^13^ and fluorescent beads^14^,^15^, allow to highlight areas of high flow through the TM, but either have cytotoxic effects^16^ or do not easily permit the examination of elements downstream of the TM.

The purpose of this study was to develop a well calibrated, quantitative, differential canalography technique to assess conventional outflow enhancement. We hypothesized that this method could highlight outflow enhancement obtained by TMB and AIT. We developed a MIGS training model using enucleated pig eyes.

## Results

**Figure 1:**
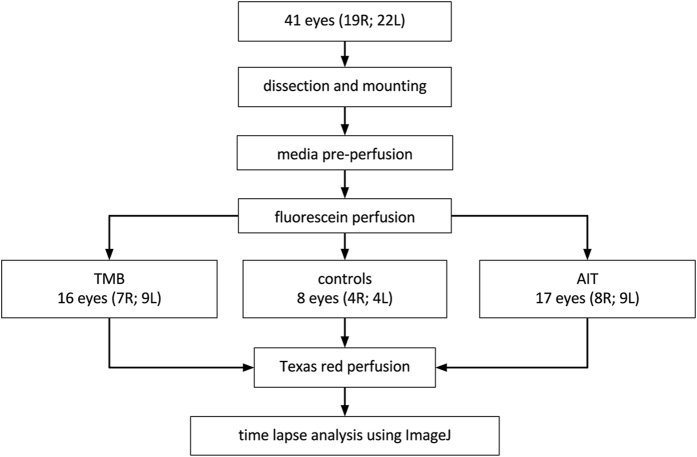
Group assignment. Flowchart detailing assignment to controls, trabecular micro-bypass (TMB) implantation or trabectome-mediated ab interno trabeculectomy (AIT).

**Figure 2:**
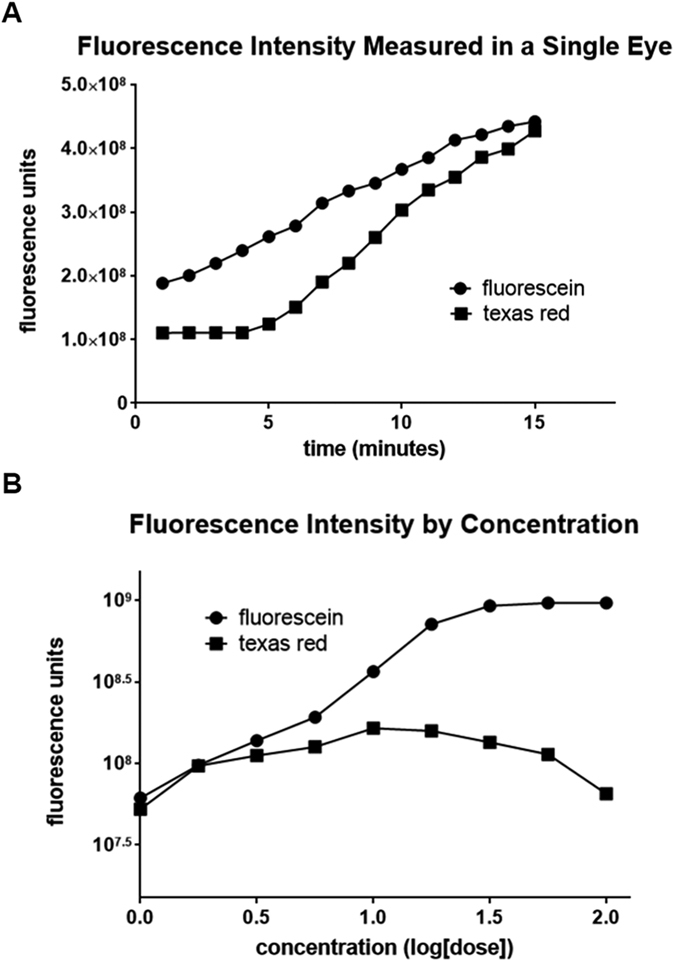
Fluorescein and Texas red fluorescence intensity and concentrations. (**A**) Texas red demonstrated a steeper increase of fluorescence intensity than fluoroscein. (**B**) Testing of logarithmic concentrations of fluorescein and Texas red indicated that the concentrations of dyes used for the experiments (concentration = 1.0) did not fall under the range of dynamic quenching.

Canalograms could be obtained in 41 out of 42 eyes (Fig. 1). Collector channels of the outflow network could be readily visualized using a new two-dye reperfusion technique. The initial filling times for fluorescein (FU) and Texas red (TR) were measured in pilot eyes to determine the proper dye sequence. These times were not significantly different (p = 0.06, n = 12). A normalization coefficient corrected TR values to match FU at select time points, thereby allowing the comparison of flow rates before and after each intervention. FU demonstrated an average increase of 56 ± 8% fluorescence units compared to TR over 4 quadrants (Supplementary Fig. S1). Eight eyes were used here to achieve >80% power (α = 0.05, two-tailed). Significant differences existed in the inferonasal (IN; p = 0.028), superonasal (SN; p = 0.048), and superotemporal (ST) quadrants (p = 0.040). The chromophore fluorescence intensities in the perilimbal region graphed over 15 minutes for each dye showed relatively linear increases in intensity over time as the dyes crossed the TM and the downstream outflow tract (Fig. 2A). The intensity slopes of TR and FU were characteristic for each dye. TR had a lower peak intensity than FU. Dyes did not exhibit chromophore quenching at the concentrations used in our experiments (Fig. 2B).

**Figure 3:**
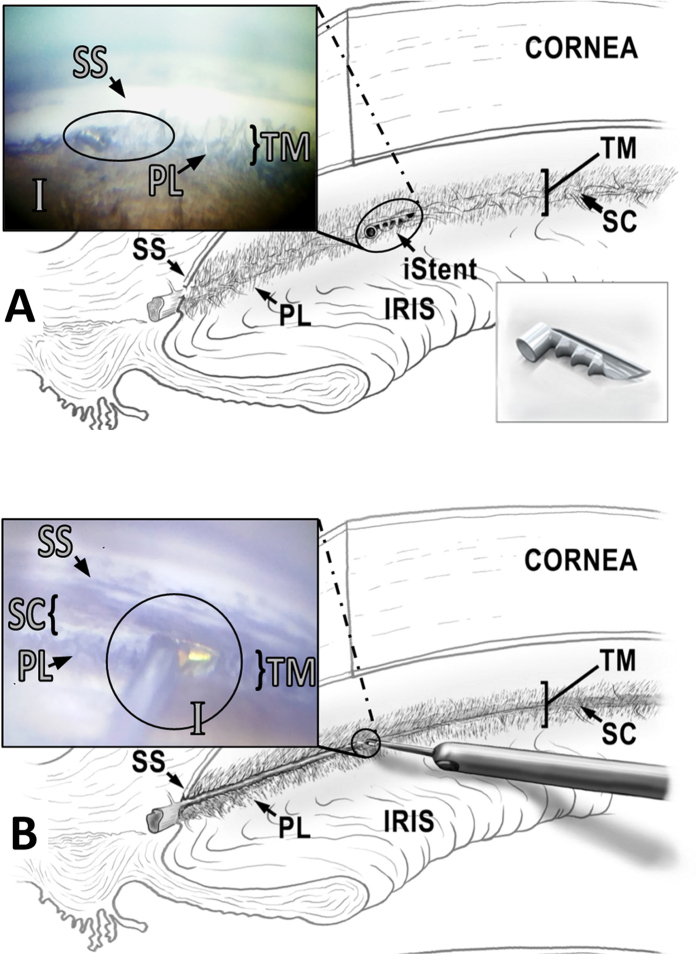
Microincisional glaucoma surgeries in pig eyes. (**A**) Intraoperative view of trabecular micro-bypass (iStent, oval) inserted through the trabecular meshwork and seated within the porcine angular aqueous plexus (distal end) with the snorkel tip parallel to the iris plane (proximal end). (**B**) Intraoperative view of ab interno trabeculectomy (Trabectome, circle). The footplate is visible immediately before trabecular meshwork engagement on the right of the tip. Meshwork has already been ablated on the left of the tip. (SS: scleral spur, TM: trabecular meshwork, I: iris, PL: pectinate ligaments, SC: Schlemm’s canal).

The angle of porcine eyes could be readily visualized by gonioscopy as done in human patients (Fig. 3). TMB implantation proceeded under gonioscopic view using the standard inserter but without viscoelastic (Fig. 3A). AIT could be performed in similar fashion and with tactile feedback that matches human eyes (Fig. 3B).

**Figure 4:**
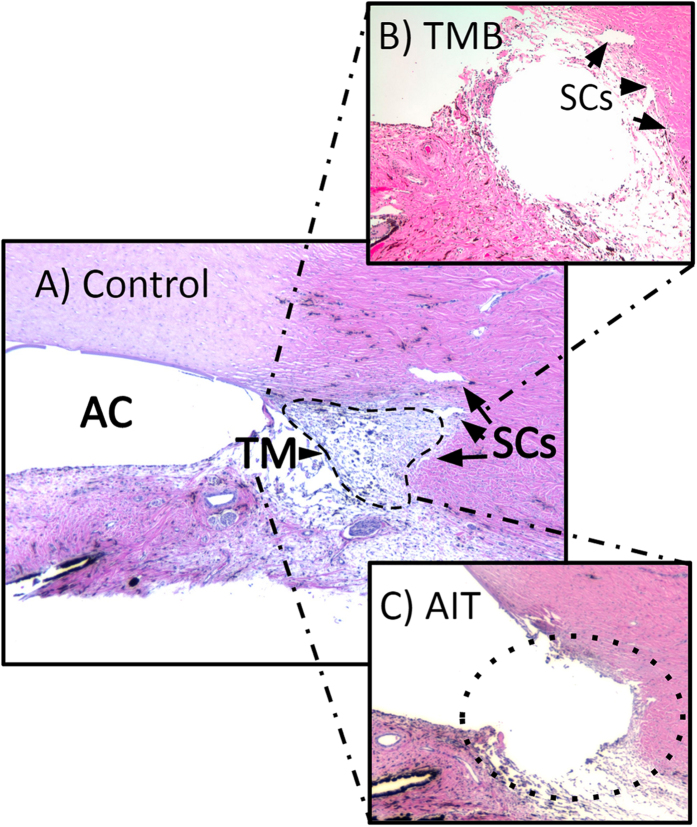
Comparison of sagittal sections of anterior chamber angle. (**A**) Control eye showing anterior chamber with trabecular meshwork (TM) and two Schlemm’s canal-like structures (SC). (**B**) Trabecular micro-bypass (TMB) implantation site is seen as a void. (**C**) Ab interno trabeculectomy (AIT) results in ablation of TM (dotted line).

Histology of the angle showed the characteristic pectinate ligaments and the large, wedge-shaped TM of porcine eyes as well as multiple Schlemm’s canal-like structures in variable locations (Fig. 4A). TMB implantation presented as a single lumen that bypassed and displaced the TM and created connections Schlemm’s canal-like structures (Fig. 4B). In contrast, AIT caused a near complete removal of the trabecular meshwork and direct connection to Schlemm’s canal-like structures (Fig. 4C). Thermal, coagulative damage to surrounding structures was mostly absent.

**Figure 5:**
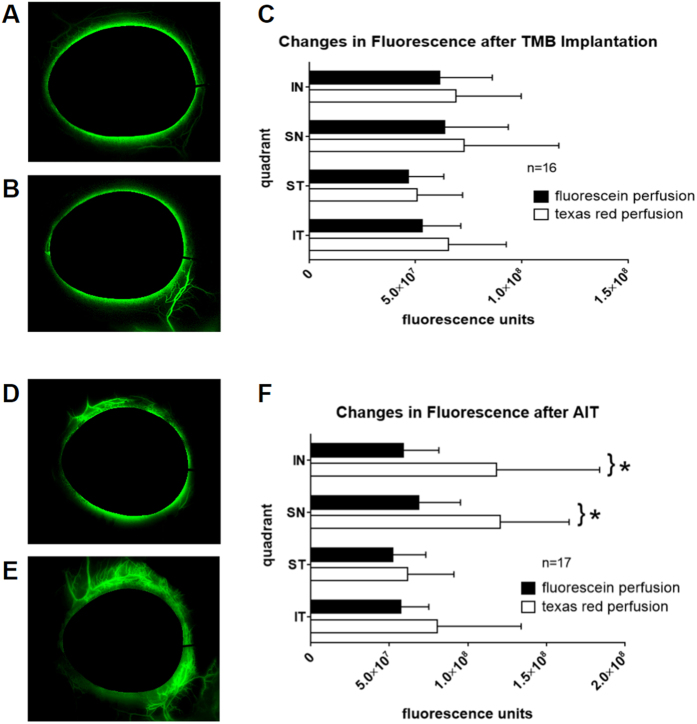
Canalograms and fluorescence intensities after TMB and AIT. Matching canalogram time points before (**A**) and after TMB (**B**), which often resulted in a confined, focal outflow enhancement near the implantation site. (**C**) Average fluorescence increases of 9–24% by quadrant (p > 0.05; IN: inferonasal, SN: superonasal, ST: superotemporal, IT: inferotemporal). Canalograms before (**D**) and after AIT (**E**), with extensive outflow enhancement beyond ablation ends. (**F**) Average fluorescence increases of 19–100% by quadrant following nasal AIT (*p < 0.05).

Time lapses of differential canalograms revealed two different outflow patterns. TMB implantation (Fig. 5A) showed a more focal outflow pattern that extended primarily radially from the location of insertion. The stented quadrant was also usually the first quadrant to show dye filling. AIT eyes (Fig. 5D) had increased flow most commonly beginning in the IN or SN, which then extended circumferentially toward centrifugal collector channels.

Comparison of canalograms before (Fig. 5A) and after TMB implantation (Fig. 5B) in 16 eyes resulted in a statistically insignificant increase of fluorescence averages by 13 ± 5%, 14 ± 8%, 9 ± 3%, and 24 ± 9% in the IN (p = 0.25), SN (p = 0.44), ST (p = 0.51), and IT (p = 0.06) quadrants, respectively (Fig. 5C). Outflow rates in these respective quadrants was calculated to be equivalent to 0.82, 0.85, 0.62, and 0.71 microliters per minute before and 0.92, 0.97, 0.68, and 0.88 microliters per minute after that procedure. AIT in 17 eyes (Fig. 5D,E) caused fluorescence intensity increases of 100 ± 50% (p = 0.0016), 75 ± 28% (p = 0.0017), 19 ± 8% (p = 0.32), and 40 ± 21% (p = 0.13) in the IN, SN, ST, and IT quadrants (Fig. 5F), respectively. Corresponding outflow rates in these quadrants increased from 0.75, 0.87, 0.66, and 0.73 to 1.49, 1.52, 0.78, and 1.02 microliters per minute, respectively.

## Discussion

The trabecular meshwork, a complex sieve-like tissue that permits fluid passage by giant vacuoles, variable pores, and transcytosis^17^, has long been considered to be the principal cause of decreased outflow in primary open angle glaucoma^9^ with most of the resistance thought to be residing in the juxtacanalicular tissue or the inner wall of Schlemm’s canal^18^,^19^. However, more recent experimental^20^ and clinical^21^ evidence suggests that a large portion of this resistance is located further downstream. Disruption^22^ or ablation of TM^4^,^21^ would be expected to achieve an intraocular pressure close to that of episcleral venous pressure around 8 mmHg^23^ but this is rarely the case^3^ and failure rates vary considerably from study to study^1^,^3^,^21^,^24^.

Although the procedures discussed here are considered minimally invasive, they are difficult to learn because they are performed on a scale that is approximately 200-fold smaller than that of traditional glaucoma surgery. Maintaining visualization during these procedures is difficult as they require concurrent movement of a surgical goniolens in one hand and MIGS applicators or ablation hand pieces in the other. Because the target tissue, the trabecular meshwork, is in very close proximity to highly vulnerable and well-vascularized structures such as the ciliary body band and iris root, novice surgeons can produce serious complications without adequate practice. To address the absence of a microincisional glaucoma surgery model, we created an *ex vivo* porcine eye system^25^ that can also be used to quantify how much outflow improvement was achieved by the trainee using a dye infusion technique^25^,^26^. We found that pig eyes provide a highly realistic and inexpensive practice environment that can serve to hone skills before first surgeries in patients. In our experience, human donor eyes rejected for use in corneal transplantation do not provide sufficient corneal clarity for learning or mastering angle surgery, and are too expensive to allow large numbers. This training model is a powerful preparation tool and does not have to be limited to the procedures discussed here but can also help to master scaffold devices^27^, ab interno sub-Tenon stents^28^, or suprachoroidal shunts^29^, as we can confirm.

Porcine eyes are well suited for this model because they share many features that are similar to human eyes but are lacking in other non-primate eyes^30^: overall size and shape are comparable; they have a large, wedge-shaped TM that allows TMB and AIT to be performed under the required, direct gonioscopic visualization^1^; possess circumferential drainage segments within the *angular aqueous plexus*^31^ that are considered analogous to Schlemm’s canal^30^,^32^,^33^. A quantitative morphometric analysis of the human versus the porcine outflow tract highlighted the anatomic similarities: human eyes have a Schlemm’s canal with depths varying from 10–25 microns and widths of 200 to 400 microns, while the angular aqueous plexus segments of porcine eyes measure 5 to 30 microns deep and 15–150 microns wide^30^. As we recently described, the Schlemm’s canal like segments in the pig are typically functionally connected and allow circumferential flow^25^. The porcine outflow^34^,^35^ tract displays biochemical glaucoma markers^32^ and giant vacuoles^36^ as seen in human eyes.

In order to provide a technique to compare local outflow enhancement from TMB and AIT, we developed a two dye perfusion technique that allows to compare pre- and postsurgical function. The trabecular meshwork and the outer wall of Schlemm’s canal are impermeable to many larger molecules or particles but can be easily passed by water-soluble fluorescent dyes. We selected the organic fluorophores TR and FU because they are readily available and have a very favorable toxicity profile^37^. Spectral domain optical coherence tomography has also recently been used to visualize the aqueous spaces of Schlemm’s canal, the collector channels^38^, and the intrascleral venous network^15^ yet this method does not allow to determine actual flow. Gold nanorods can be used as a Doppler contrast agent to estimate flow^16^ but this method is limited by inflammation^39^.

We had to use a second dye that is different from the first one in postsurgical canalograms because molecules that can flow through the TM may also eventually diffuse into the interstitial space after some time and wash out incompletely. To ensure a valid comparison of pre- and postprocedural canalograms, we thoroughly tested both dyes to account for their characteristic properties. A 19% delay in TR initial filling time in pilot experiments was sufficient evidence for using FU followed by TR for all of the eyes. Choosing this order avoided false positive flow enhancement after the surgical procedures. TR has a molecular weight of 625 g·mol^−1^, almost twice of the molecular weight of FU (332 g·mol^−1^). The two dyes revealed different baseline fluorescence intensities and slope magnitudes, thus resulting in variations in fluorescent intensities at similar time points within both time lapses. Although the relationship between dye concentration and fluorescence intensity is initially linear^40^, very high concentrations of these dyes can result in a decrease of fluorescence intensity as a result of dynamic quenching, an effect described by the Stern-Volmer equation in which excited chromophore molecules will interact with each other and lose energy through processes other than fluorescent emission^41^. Our testing of logarithmic concentrations of these dyes and measuring their emitted fluorescence through ImageJ ensured that the concentrations used for FU and TR here did not exhibit dynamic quenching. A normalization coefficient corrected TR values to match FU thereby allowing a direct comparison of flow rates before and after each intervention^42^,^43^.

The purpose of this study was to developed an *ex vivo* training system that allows new surgeons to practice in a safe and highly realistic environment. We also wanted to create a differential, quantitative canalography method that uses slope-adjusted fluorescence intensities (to avoid quenching seen at high intensities) of two different chromophores to better highlight outflow enhancements obtained by TMB and AIT. Although outflow patterns of the two MIGS modalities examined here are different, it is important to recall that circumferential flow is more restricted in this pig eye model due to segmentation of the perilimbal outflow tract and that these results do not represent the performance in human eyes. The TMB is designed for insertion into a single lumen human Schlemm’s canal while in the pig placements may be more commonly imperfect and only partially into Schlemm’s canal like segments. Another limitation concerns the size of the aqueous plexus segments themselves. These segments can have a smaller width than the lumen of a singular human Schlemm’s canal^30^. It is possible that the intracanalicular portion of the TMB stents may at least be partially obstructed by tissue between segments of the aqueous plexus or the outer wall itself, a challenge that can be observed in histological sections of human eyes as well^1^. Similarly, although TMBs were implanted under direct visualization with confirmed placement, small, unintended variations in technique may impact placement and performance in this porcine model more than in human eyes. Therefore, conclusions about the clinical performance of either TMB or AIT in human eyes based on the results presented here cannot be extrapolated.

The amount of circumferential flow likely correlates with the number of drainage segments that a procedure can access effectively. A single point of access to the outflow tract, as delivered by TMB, is thought to enable flow over approximately 60 degrees in human eyes^7^ and might be considerably less here. In contrast, AIT can ablate TM over up to 180 degrees in experienced hands thereby providing access to 180 plus 60 degrees, totaling to 240 degrees of outflow segments^1^. We limited ablation in this study to 90 degrees of the nasal angle because this amount is achievable by most surgeons with ease. It is important to keep in mind that despite differences in amount of angle access and high versus low immediately achieved outflow enhancement, little is known about long-term changes of the outflow system or wound healing and foreign body reaction to microimplants.

In conclusion, we present an *ex vivo* training model for microincisional glaucoma surgery in pig eyes. We introduce a new differential canalogram technique that controls for dynamic quenching of two different chromophores, and allows for quantification of outflow enhancement after trabectome-mediated ab interno trabeculectomy and trabecular micro-bypass in this species.

## Methods

### Preparation and Pre-Perfusion of the Eyes

Correctly paired, enucleated porcine eyes from a local abattoir were processed within two hours of sacrifice. Each eye was identified as left or right and copiously irrigated with phosphate buffered saline (PBS, Thermo Fisher Scientific, Waltham, MA). Extraocular tissues were trimmed to the length of the globe. Eyes were placed on cryogenic vial cups (CryoElite Cryogenic Vial #W985100, Wheaton Science Products, Millville, NJ) to encompass the optic nerve in a compression-free mount. A 30-gauge needle was inserted through the nasal cornea approximately 2 mm anterior to the limbus parallel to the iris and advanced to the center of the anterior chamber with the bevel up. Eyes were gravity perfused with 37 °C clear Dulbecco’s Modified Eagle’s Media (DMEM, Hyclone, GE Healthcare Life Sciences, Piscataway, NJ, USA) for 15 minutes with the pressure set to the regular porcine intraocular pressure of 15 mmHg^30^ as used in anterior chamber perfusion systems^42^,^43^,^44^.

### Chromophore Analysis

When incomplete dye washout was observed in single dye reperfusion pilot experiments, two chromophores with different peak excitation wavelengths (fluorescein (FU): 494 nm, Texas red (TR): 589 nm) were used. The proper detection sensitivities for those were determined using a hemocytometer chamber filled with 10 μL of each dye in clear DMEM at concentrations of 0.0332 mg/mL and 0.28 mg/mL, respectively. Identical average gray values on a 14 bit image were obtained at exposures of 15 and 10 milliseconds, respectively.

Six eyes were first perfused with FU (AK-FLUOR 10%, Fluorescein injection, USP, 100 mg/ml, NDC 17478-253-10, Akorn, Lake Forest, IL) followed by TR (sulforhodamine 101 acid chloride, 10 mg, Alfa Aesar, Ward Hill, MA) to establish whether the order of the dyes would affect the perfusion rate or the intensity of fluorescence; another six eyes underwent the reverse order. Initial filling time, recorded as the time at which the dyes could first be observed entering the proximal outflow tract structures (the perilimbal regions), were recorded for each eye quadrant using ImageJ (ImageJ 1.50b, http://imagej.nih.gov/ij, Wayne Rasband, National Institutes of Health, Bethesda, MD). Eight further control eyes (4 left, 4 right) were run with FU and TR with no intervention in between. After FU and TR time lapse analyses were performed at each eye’s respective half-maximum FU intensity frame, fluorescence units were compared in all four quadrants.

Next, FU and TR were sequentially perfused in a single eye with time lapses (CellSens, Olympus Life Science, Tokyo, Japan) taken as described below. Raw fluorescent intensities of the perilimbal flow patterns were collected every minute for a total of 15 minutes for each dye. When the canalograms of the control eyes revealed consistency between the two chromophores, a normalization coefficient (*c* = 1.56) was computed to adjust TR to match FU at a single relative time point in each time lapse pair, allowing for a comparison of flow rates before and after each intervention.

We then determined the fluorescent intensities for FU and TR in each eye at the same relative time points of half-maximum fluorescence of FU as measured in ImageJ. This kept the time factor constant and allowed to compare outflow rates per quadrant in microliters per minute. For this computation, the aqueous humor flow rate of three microliters per minute of porcine eyes was divided by the relative fluorescence of each quadrant measured in control eyes and used as the baseline to compare postsurgical flow.

### Differential Canalograms

The fluorescent tracer reperfusion technique was used in 41 eyes to quantify outflow changes from TMB implantation or AIT. Whole pig eyes were prepared, mounted, and pre-perfused with DMEM as described above. Fluorescein was then gravity-infused at a concentration of 0.0332 mg/ml in clear DMEM for 15 minutes. The chromophore flow pattern was recorded as a time lapse using a stereo dissecting microscope (Olympus SZX16 and DP80 Monochrome/Color Camera; Olympus Corp., Center Valley, PA) equipped for fluorescent imaging (Chroma 49002 GFP cube and Chroma 49004 DSRED cube, Chroma Technology Corp, Bellow Falls, VT). En face images were obtained every 20 seconds at 580 × 610 pixel resolution with 2 × 2 binning and 14 bit depth (CellSens, Olympus Life Science, Tokyo, Japan). FU infusion was then stopped and the needle removed. A surgeon experienced in microincisional glaucoma surgery (NAL) performed all AITs and TMB implantations as described below (Fig. 3). The temporal, clear corneal incision was sealed in a watertight fashion using cyanoacrylate. Clear DMEM containing TR at a concentration of 0.28 mg/ml was infused for 15 minutes with a time lapse recorded. At conclusion, the eyes were processed for histology. TMB eyes were marked at the site of implantation and the stent was removed. All eyes were rinsed in PBS, hemisected, and fixed with 4% paraformaldehyde at room temperature followed by PBS for 48 hours before being placed in 70% ethanol. A corneoscleral wedge from the surgical site was paraffin-embedded for histological processing, cut at 10 μm thickness, and stained with hematoxylin and eosin (H&E).

### Trabecular Meshwork Bypass Implantation Technique

Sixteen pig eyes underwent TMB implantation (iStent, Glaukos Corporation, Laguna Hills, CA, USA). The surgical technique was analogous to that used in human eyes^45^. With the temporal side of the eye facing the surgeon, a clear corneal incision was created 2 mm anterior to the temporal limbus with a 1.8 mm keratome. The loaded TMB applicator was inserted into the anterior chamber and advanced toward the TM. The stent was driven through the TM using a gentle sweeping motion. Only the proximal end of the stent remained visible in the anterior chamber (Fig. 3A). The stent was ejected from the applicator and the applicator tip was removed. A drop of cyanoacrylate was used to seal the temporal, clear corneal incision.

### Ab Interno Trabeculectomy Technique

After infusion with FU, 17 eyes underwent AIT, performed analogous to AIT in human eyes^1^. Eyes were positioned under a surgical microscope with the temporal side directed toward the surgeon. A 1.8 mm keratome was used to create a clear corneal incision 2 mm anterior to the temporal limbus. The inner third was slightly flared to improve mobility and eliminate striae from torque. The eyes were then tilted by 30 degrees toward the nasal side and a goniolens (Goniolens ONT-L, #600010, NeoMedix Inc., Tustin, CA) was placed on the cornea to visualize the nasal chamber angle. The tip of the trabectome handpiece was inserted into the anterior chamber with constant irrigation, and gentle goniosynechiolysis with the smooth base plate was performed to disinsert pectinate ligaments (Fig. 3B). The TM was engaged and Schlemm’s canal entered with a left and upward movement. TM ablation at 1.1 mW ensued toward the left for 45 degrees with appropriate rotation of the goniolens to maintain visualization. The tip was then disengaged from the TM, rotated 180 degrees within the eye, and positioned at the original starting location. A 45 degree ablation was performed towards the right. The handpiece was removed, and the temporal, clear corneal incision closed watertight with a drop of cyanoacrylate.

### Time Lapse Analysis

Individual time lapses with FU and TR were analyzed using ImageJ software^46^,^47^. For each fluorescein time lapse, the half-maximum perilimbal fluorescence was calculated, and the appropriate frame containing perilimbal fluorescence that best matched that value was selected for analysis. The same frame number was used in the corresponding Texas Red time lapse for each eye. A masked observer measured raw fluorescence intensities from these frames by outlining the quadrants containing fluorescent outflow channels. Each pig eye was divided into four quadrants: inferonasal (IN), superonasal (SN), superotemporal (ST), and inferotemporal (IT). Quadrant outlines began at the limbus of each quadrant to exclude quantification of fluorescence of the dye in the anterior chamber.

### Statistics

Student’s paired two sample t-test was used to compare outflow changes in the same eyes before and after each intervention. The unpaired t-test was utilized to detect any significant differences between right and left eyes. A sample size of at least seven eyes was calculated as the minimum number of control eyes needed to detect a significant difference between the two-dye reperfusion technique with 80% statistical power and an alpha error of 0.05 on a two-tailed matched pair comparison. Similarly, pilot data in the experimental groups revealed a sample size of at least 16 eyes in each group to reliably detect significant changes before and after each intervention using the same test parameters for power and alpha error.

## Additional Information

**How to cite this article**: Parikh, H. A. *et al*. Differential Canalograms Detect Outflow Changes from Trabecular Micro-Bypass Stents and Ab Interno Trabeculectomy. *Sci. Rep.* **6**, 34705; doi: 10.1038/srep34705 (2016).

**Publisher’s note:** Springer Nature remains neutral with regard to jurisdictional claims in published maps and institutional affiliations.

## Supplementary Material

Supplementary Information
