# Supplementary material for: Differential Canalograms Detect Outflow Changes from Trabecular Micro-Bypass Stents and Ab Interno Trabeculectomy
Source: Sci Rep. 2016 Nov 4;6:34705. doi: 10.1038/srep34705 (PMC5095709; doi:10.1038/srep34705)
Supplement: Supplementary Information [file srep34705-s1.pdf]

Supplementary Information

Differential Canalograms Detect Outflow Changes from Trabecular Micro-Bypass Stents and Ab Interno Trabeculectomy

Hardik A. Parikh[1, 2], Ralitsa T. Loewen[1], Pritha Roy[1], Joel S. Schuman[1, 3, 4], Kira L. Lathrop[1,4], Nils A. Loewen[1*]

[1]Department of Ophthalmology, University of Pittsburgh School of Medicine, Pittsburgh, PA 15213, United States
[2]New Jersey Medical School, Rutgers State University of New Jersey, Newark, NJ 07103, United States
[3]Department of Ophthalmology, New York University School of Medicine, NY 10016, United States
[4]Department of Bioengineering, University of Pittsburgh Swanson School of Engineering, Pittsburgh, PA 15261, United States

*corresponding author

Figure S1.

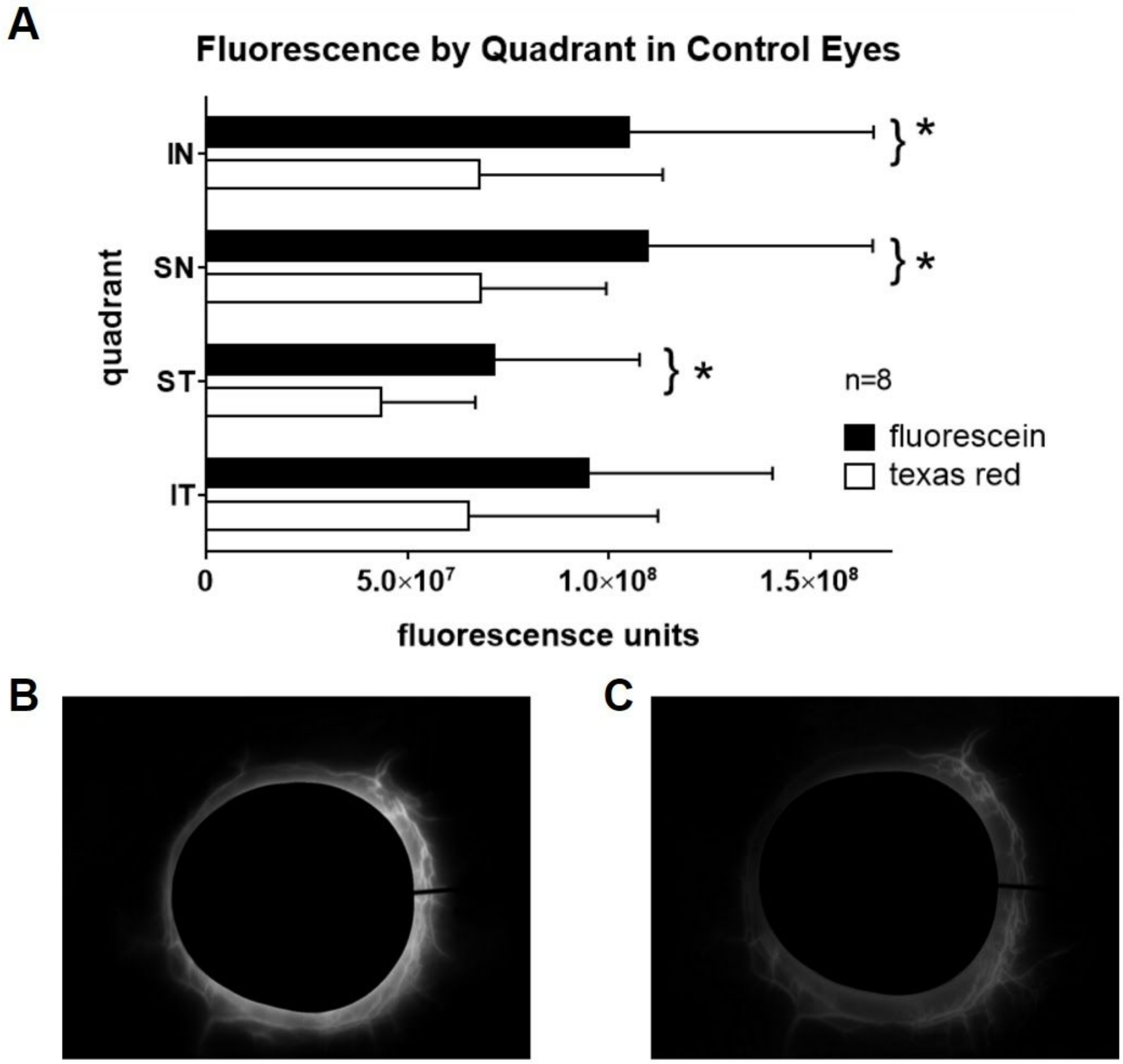

Figure S1. Fluorescein and Texas red fluorescence intensities for normalization coefficient. Fluorescence intensities were compared in precisely paired four right and four left control eyes to compute the normalization coefficient. A) Mean fluorescent intensities of fluorescein were higher than Texas red in each quadrant by: 55% in IN, 61% in SN, 64% in ST, and 46% in IT. The differences were statistically significant in IN (p=0.028), SN (p=0.048), and ST (p=0.040). Single frames were captured at the time of half-maximum perilimbal fluorescence of the fluorescein (B) canalogram. The Texas red (C) canalogram was captured at the same relative time point in the same control eye. Unadulterated images shown as grey images demonstrated the dimmer fluorescence intensity of Texas red.
